# A comprehensive comparison of single- and composite-seaweed feeds on growth performance, lipid metabolism, and immune function of large yellow croaker (*Larimichthys crocea*)

**DOI:** 10.3389/fcimb.2026.1947273

**Published:** 2026-10-06

**Authors:** Yueming Dang, Liyue Chai, Ruochen Xia, Sifeng Zhu, Guoyi Li, Rui Yang, Jianping Wang, Juanjuan Chen, Qiqin Liu

**Affiliations:** 1 School of Marine Sciences, Ningbo University, Ningbo, China; 2 Ningbo Academy of Oceanology and Fisheries, Ningbo, China

**Keywords:** composite seaweed feed, growth performance, immune, Larimichthys crocea, lipid composition

## Abstract

**Introduction:**

The large yellow croaker (*Larimichthys crocea*) is an economically important fish species in China, yet its quality and value are compromised by excessive fat deposition induced by high-fat diets under high-density aquaculture. Our previous study noted the lipid-regulating potential of individual seaweed components like fucoidan and kelp (*Saccharina japonica*) powder, the synergistic effects of composite seaweed feeds remain underexplored.

**Methods:**

This study investigated the effects of 3% *Neoporphyra haitanensis* compared with its control feed (CM1), and two composite seaweed feeds (FNKM1: 1% kelp + 1% *N. haitanensis* + 0.1% fucoidan; FNKM2: 2% kelp + 2% *N. haitanensis* + 0.2% fucoidan) compared with the control feed (CM2) over an 8-week feeding trial.

**Results and discussion:**

The results showed that single *N. haitanensis* supplementation, despite limited growth promotion, improved muscle lipid composition by reducing triglycerides (TG) and LDL-C contents while increasing beneficial phospholipids. Notably, the composite seaweed feeds, particularly FNKM2, showed more pronounced effects on growth and lipid-related parameters, with WGR reaching 1.66-fold, accompanied by a 1.33-fold increase in HDL-C and a 24.20% decrease in crude lipid content. In addition, the composite seaweed diets (FNKM1 and FNKM2) significantly downregulated most of the differentially abundant lipids in muscle. Among these, TG and phospholipids were significantly reduced in the FNKM1 group. These changes were accompanied by the upregulation of fatty acid oxidation genes (*cpt1*, *ppara*, *pgc-1a*) and the TG hydrolysis-related gene (*lpl*). Both feeds also enhanced immune-antioxidant capacity (∑IoI >1): FNKM1 significantly increased ACP, ALP, lysozyme, GSH-Px, and CAT activities, whereas FNKM2 increased ACP, ALP, and CAT activities. Both groups modulated hepatic cytokine expression, including *il-4*, *il-1b*, and *tnf-a*. Overall, the composite seaweed feeds contributed to improved growth, optimized lipid metabolism, and enhanced immune function in large yellow croaker, offering a scientific basis for functional feed development in intensive aquaculture.

## 1 Introduction

The large yellow croaker (*Larimichthys crocea*) is an economically important marine fish species in China, with an annual yield exceeding 280, 000 tons (The People’s Republic of China Ministry of Agriculture, Fisheries Burea, 2024). It is popular with consumers because of its golden yellow body and abundant nutrient contents. In response to declining wild yellow croaker resources combined with continuous growth in market demand, intensive aquaculture modes using cages or enclosures have become major sources of large yellow croaker supply (Lv et al., 2024). However, long-term feeding with artificially formulated feed under high-density aquaculture conditions often leads to excessive body fat accumulation and tender flesh, potentially even inducing lipid metabolism abnormalities, oxidative stress, and inflammatory responses, which severely limits the edible quality and market value of large yellow croakers (Ji et al., 2021; Li et al., 2021; Naiel et al., 2023). Therefore, regulating lipid metabolism through feed nutrient supplementation strategies has become critical for improving the healthy aquaculture and quality of large yellow croakers (Fan et al., 2021; Liu et al., 2024).

Seaweed is a natural functional feed additive that is rich in polysaccharides, carotenoids, vitamins, minerals, and other bioactive substances. Seaweed has demonstrated good potential in improving lipid metabolism, body color, and meat quality in fish (Ijaola et al., 2024). Kelp (*Saccharina japonica*) and *Neoporphyra haitanensis*, two economically important seaweed species that produce the highest seaweed yields and market value in China, possess both edible and medicinal value. These seaweed species have been shown to promote metabolism, regulate cholesterol, boost immunity, and exert antioxidant effects (He et al., 2023; Yue et al., 2022; Zuo et al., 2023). Previous studies indicate that kelp and its active ingredient fucoidan can effectively regulate lipid composition and cholesterol metabolism in large yellow croaker, in addition to improving gut health and enhancing immunity (Liu et al., 2024; Zhao et al., 2021; Zhu et al., 2025). Fucoidan has been shown to significantly decrease serum triglycerides and total cholesterol levels in yellow catfish (*Pelteobagrus fulvidraco*) fry (Yang et al., 2014). In addition, a study on Pacific white shrimp (*Litopenaeus vannamei*) found that supplementation with *N. haitanensis* powder could significantly boost growth, increase the levels of polyunsaturated fatty acids such as docosahexaenoic acid (DHA) and eicosapentaenoic acid (EPA), and promote immunity and antioxidant capacity (Shen et al., 2024). *N. haitanensis* has also been shown to inhibit lipid accumulation in serum, liver, and adipose tissue and regulate gut microbiome homeostasis in obese mice (Wang et al., 2022). These reports suggest that seaweed and its active ingredients exhibit good application potential as functional feed additives.

Most studies in this area focus on the additive effects of a single type of seaweed. However, seaweed components and functional limitations may influence the results in actual application. In recent years, the synergistic effects of composite seaweed additives have attracted increasing attention. Many studies have demonstrated that utilizing a combination of different algal species and their active ingredients was a significantly better approach than the use of a single seaweed source in terms of improving growth performance, increasing stress and disease resistance, optimizing gut health, and regulating liver lipid metabolism in aquaculture animals (García-Márquez et al., 2023; Sattanathan et al., 2023; Seyedalhosseini et al., 2023; Zhang et al., 2025). For example, Ferreira found that supplementation with *Gracilaria gracilis* and *Nannochloropsis oceanica* as feed additives could decrease the gut microbial diversity of European seabass (*Dicentrarchus labrax*), while a combination of both could alleviate this detrimental effect (Ferreira et al., 2022). Furthermore, Seong et al. demonstrated that the combined addition of *Nannochloropsis* and *Schizochytrium* powder could significantly increase the growth performance of red sea bream (*Pagrus major*), and further improvements were obtained after supplementation with *Chlorella* powder (Seong et al., 2021). These results indicate that rational combinations of algal species can achieve active ingredient complementation, thereby producing synergistic effects.

Although composite seaweed supplementation strategies (e.g., combining red alga *Gracilaria pulvinata* and brown alga *Sargassum ilicifolium* powder in equal proportions) have demonstrated advantages in aquaculture, the specific combination of kelp, *N. haitanensis* or their bioactivity components has not been systematic evaluated (Seyedalhosseini et al., 2023). In the present study, we evaluated the beneficial effects of single supplementation with 3% *N. haitanensis* powder on the growth performance and lipid composition of large yellow croakers. To further determine the synergistic effects of composite seaweed feed, we formulated composite seaweed feed containing different concentrations of kelp, fucoidan, and *N. haitanensis* powder. Specifically, the differences between single and composite seaweed feed in regulating large yellow croaker growth, lipid metabolism, and immunity were investigated. Our finding provides new strategies for developing functional feed that can alleviate lipid deposition in large yellow croakers.

## 2 Materials and methods

### 2.1 Feed preparation

The experimental diets and feeding procedures for large yellow croaker were conducted following the methods described by Liu et al. (2024). A commercially available feed from Zhejiang Qiangpu Biotechnology Co., Ltd. (China) served as the control diet (CM1 and CM2 groups). In addition to the basal control formulation, the NM group diet was supplemented with 3% *N. haitanensis* powder. Furthermore, two composite seaweed-derived diets were formulated: FNKM1, containing 1% kelp, 1% *N. haitanensis* and 0.1% fucoidan; and FNKM2, containing 2% kelp, 2% *N. haitanensis*, and 0.2% fucoidan. The formulation and proximate composition of the feeds were presented in **Table 1**. Fresh kelp and *N. haitanensis* were obtained from Xiangshan Xuwen Seaweed Development Co., Ltd. (China) and Ningbo Yinzhou Tengye Seaweed Co., Ltd. (China), respectively. The seaweeds were dried, ground and passed through a 100-mesh sieve to obtain seaweed powders. Fucoidan (purity >85%) was sourced from Shandong Jiejing Group Co., Ltd. (China). All the feeds were pelleted, dried at 50 °C, and subsequently stored at –20 °C until use to maintain nutrient stability and prevent degradation.

**Table 1:** Formulation and proximate compositions of feed (% dry weight, n = 3).

Ingredients	CM1	NM	CM2	FNKM1	FNKM2
Fish meal	38	38	38	38	38
Chicken powder	5	5	5	5	5
Soy protein concentrate	9	9	9	9	9
Soybean meal	14.5	14.5	14.5	14.5	14.5
Corn protein powder	5	5	5	5	5
High gluten flour	21	18	21	18.9	16.8
Fish oil	2	2	2	2	2
Soybean oil	2	2	2	2	2
Ga(H_2_PO_4_)_2_	1	1	1	1	1
Lutein	0.3	0.3	0.3	0.3	0.3
Choline chloride	0.1	0.1	0.1	0.1	0.1
Vitamin premix	0.5	0.5	0.5	0.5	0.5
Mineral elements	1.5	1.5	1.5	1.5	1.5
Fucoidan	0	0	0	0.1	0.2
Kelp powder	0	0	0	1	2
*N. haitanensis* powder	0	3	0	1	2
Proximate compositions (% dry weight)					
Protein	48.82 ± 2.44^a^	49.89 ± 3.15^a^	48.49 ± 2.29^a^	48.04 ± 2.77^a^	47.84 ± 2.77^a^
Lipid	12.23 ± 1.60^a^	11.71 ± 1.38^a^	13.89 ± 1.32^a^	13.20 ± 1.56^a^	12.74 ± 1.61^a^
Ash	11.02 ± 1.54^a^	11.14 ± 1.89^a^	13.10 ± 2.03^a^	13.44 ± 2.16^a^	13.60 ± 2.48^a^
Moisture	7.51 ± 0.86^a^	6.89 ± 0.81^a^	7.13 ± 0.64^a^	7.13 ± 0.76^a^	7.21 ± 0.92^a^

Proximate composition data are presented as mean ± SD with three replicates (n = 3). CM1, CM2 groups: control group fed with the commercial feed; NM group: single seaweed group fed with feed supplemented with 3% *Neoporphyra haitanensis* powder; FNKM1 group: composite seaweed group fed with feed supplemented with 1% kelp, 1% *N. haitanensis*, and 0.1% fucoidan; FNKM2 group: composite seaweed group fed with feed supplemented with 2% kelp, 2% *N. haitanensis*, and 0.2% fucoidan. One-way ANOVA was used to compare all groups, and *p* < 0.05 was considered statistically significant. The superscript letters indicate significant differences between the compared groups.

### 2.2 Experimental procedure

The trial with *N. haitanensis*-supplemented diets (CM1, NM) ran from May to July 2023, and that with composite seaweed diets (CM2, FNKM1, FNKM2) from June to August 2024. Both experiments were conducted at Ninggang Aquaculture Co., Ltd., Xiangshan County, Ningbo City, Zhejiang Province, China (29°32’36” N, 121°46’23” E). Before each trial, fish were acclimatized for two weeks in floating sea cages (12.0 m × 12.0 m × 8.0 m) and fed a basal diet during this period. At the beginning of the formal trial, healthy large yellow croakers with average body weights of 200.00 ± 16.8 g (the CM1 and NM groups) and 213.25 ± 14.77 g (the CM2, FNKM1, and FNKM2 groups), were selected. All fish were free of apparent abnormalities or deformities. Following 24 h of fasting, the fish were randomly stocked into floating sea cages (4.0 m × 4.0 m × 5.0 m) at a density of 560 individuals per cage, with three replicate cages assigned to each dietary treatment. The fish were fed twice daily (05:00 and 17:00) at a daily ration of 1%–3% of their body weight. During the experimental period, water quality parameters were recorded as follows: for the CM1 and NM groups, seawater temperature ranged from 19 °C to 27 °C, salinity from 28 to 33, and dissolved oxygen (DO) from 5 to 6 mg/L; for the CM2, FNKM1, and FNKM2 groups, temperature was 27 °C–31 °C, salinity 27–31, and DO 7–8 mg/L.

### 2.3 Sample collection

At the end of the 8-week feeding trial, fish were fasted for 24 h and anesthetized with eugenol (1:10, 000, w/v; Sinopharm Chemical Reagent, China). For each cage, 10 fish were randomly sampled (30 fish per feed treatment) for measurements of body weight, body length, body height, caudal peduncle length, and caudal peduncle height. These measurements were used to determine the body length/height, caudal peduncle length/height, weight gain rate (WGR), specific growth rate (SGR) and condition factor (CF). Of these, 3 fish from each cage were further randomly selected (9 fish per feed treatment) for dissection and collection of viscera and liver to calculate the viscerosomatic index (VSI) and hepatopancreas index (HSI). Blood, dorsal muscle, and abdominal muscle tissues were also collected from these 9 fish, with tissues from the three fish within each cage were pooled as one biological replicate, yielding three replicates per dietary group. Blood samples were temporarily stored at 4 °C and then transferred to the laboratory for further preparation of serum, which was used for immune- and antioxidant-related enzyme activity measurements. Liver tissues were analyzed for inflammatory cytokine gene expression. A mixture of dorsal and abdominal muscle was used to determine crude fat, cholesterol content, as well as the expression of lipid metabolism-related genes. For lipidomic analysis, two of the three fish sampled from each cage were randomly selected, and dorsal and abdominal muscle tissues from each individual fish were pooled and analyzed as one biological replicate (n = 6 per feed treatment). Liver and muscle samples were immediately frozen in liquid nitrogen and stored at −80 °C until further analysis.

### 2.4 Determination of serum immune-related enzyme activities

Blood samples maintained at 4 °C and subsequently centrifuged at 5000 × g for 10 min at 4 °C to obtain serum. Activities of serum immune- and antioxidant-related enzymes, including acid phosphatase (ACP), glutathione peroxidase (GSH-Px), superoxide dismutase (SOD), lysozyme (LYS), alkaline phosphatase (ALP), and catalase (CAT), were measured using commercial assay kits (Nanjing Jiancheng Bioengineering Institute, China) according to the manufacturer’s instructions (catalog numbers: ACP, A060-2-1; GSH-Px, A005-2-1; SOD, A001-1-2; LYS, A050-1-1; ALP, A059-2-2; CAT, A007-1-1). Absorbance was read using a Thermo Varioskan Flash microplate reader (Thermo Fisher Scientific, USA).

### 2.5 RNA extraction and real-time quantitative PCR

Liver and muscle tissues were used for the analysis of inflammatory cytokine genes, including interleukin-1β (*il-1β*), interleukin-6 (*il-6*), interleukin-8 (*il-8*), interleukin-10 (*il-10*), tumor necrosis factor-α (*tnf-α*), and interferon-γ (*ifn-γ*), and lipid metabolism-related genes, including acyl-CoA oxidase (*aco*), carnitine palmitoyltransferase 1 (*cpt1*), fatty acid synthase (*fas*), lipoprotein lipase (*lpl*), peroxisome proliferator-activated receptor α (*pparα*), peroxisome proliferator-activated receptor γ coactivator 1α (*pgc-1α*), and sterol regulatory element-binding protein 1 (*srebp1*), respectively. Total RNA was extracted using Trizol reagent (Invitrogen, USA; catalog number: 15596026). RNA concentration and purity were measured with a NanoDrop 2000 spectrophotometer (Thermo Fisher Scientific, USA), while integrity was verified by 1% agarose gel electrophoresis. Reverse transcription and RT-qPCR were performed according to Zhu et al. (2025). Melting curve analysis confirmed primer specificity, and relative gene expression was calculated using the 2^−ΔΔCT^ method, with *β-actin* as the internal reference. Primer sequences are provided in **Table 2**. Each assay was run in triplicate, and the experiment was repeated three times.

**Table 2:** Primer sequences used for real-time quantitative PCR.

Target genes	Forward primers (5’-3’)	Reverse primers (5’-3’)	NCBI accession number
reference gene			
*β-actin*	CTACGAGGGTTATGCCCTGCC	TGAAGGAGTAACCGCGCTCTGT	KM501500
Inflammatory cytokine genes in the liver			
*il-1β*	CATAGGGATGGGGACAACGA	AGGGGACGGACACAAGGGTA	KJ459927
*il-6*	CGACACACCCACTATTTACAAC	TCCCATTTTCTGAACTGCCTCT	KU140675
*il-8*	AATCTTCGTCGCCTCCATTGT	GAGGGATGATCTCCACCTTCG	KP202400.1
*il-10*	AGTCGGTTACTTTCTGTGGTG	TGTATGACGCAATATGGTCTG	XM010738826
*tnf-α*	ACACCTCTCAGCCACAGGAT	CCGTGTCCCACTCCATAGTT	EF165623
*ifn-γ*	TCAGACCTCCGCACCATCA	GCAACCATTGTAACGCCACTTA	KM501500
Lipid metabolism related genes in the muscle			
*aco*	AGTGCCCAGATGATCTTGAAGC	CTGCCAGAGGTAACCATTTCCT	JX456348
*cpt1*	GCTGAGCCTGGTGAAGATGTTC	TCCATTTGGTTGAATTGTTTACTGTCC	JX434612
*fas*	CAGCCACAGTGAGGTCATCC	TGAGGACATTGAGCCAGACAC	JX456351
*lpl*	GAGAGGATTCATCTGCTGGGTTAC	ACATCAACAAACTGGGCGTCATC	JQ327827
*pparα*	GTCAAGCAGATCCACGAAGCC	TGGTCTTTCCAGTGAGTATGAGCC	KF998577
*pgc-1α*	CTGCTCAGTATGGCAACGA	GGTCACTGGCATTGGTCAC	KM593914
*srebp1*	TCTCCTTGCAGTCTGAGCCAAC	TCAGCCCTTGGATATGAGCCT	KP342262

### 2.6 Determination of muscle crude fat and cholesterol content, and lipidomic analysis

#### 2.6.1 Crude lipid content

Crude lipid content was analyzed through Soxhlet extraction. Approximately 0.5 g of dried muscle sample was weighed into a pre-weighed filter paper thimble and placed in the extraction chamber. Petroleum ether was added via the condenser, and extraction was performed continuously at 50 °C for 8 h with a reflux rate of 6–8 cycles per hour. After extraction, the thimble was oven-dried at 60 °C to constant weight, cooled, and re-weighed. Crude lipid content (% dry weight) was calculated as (m_1_ − m_0_) / m_2_ × 100, where m_1_ is the weight of the thimble with lipid after extraction, m_0_ is the initial weight of the empty thimble, and m_2_ is the weight of the dried muscle sample (approximately 0.5 g).

#### 2.6.2 Cholesterol content

Total cholesterol (T-CHO), high-density lipoprotein cholesterol (HDL-C), and low-density lipoprotein cholesterol (LDL-C) contents in large yellow croaker muscle were determined using commercial assay kits (Nanjing Jiancheng Bioengineering Institute, China) according to the manufacturer’s protocol (catalog numbers: T-CHO, A111-1-1; HDL-C, A112-1-1; LDL-C, A113-1-1).

#### 2.6.3 Lipidomic analysis

Approximately 50 mg of muscle tissue was weighed for lipid extraction and subsequent UPLC-MS analysis following Zhu et al. (2025). Raw data were processed in LipidSearch software (v4.2.28), with retention time (RT) tolerance set to 0.25 and m-Score threshold set to 3 for peak annotation, alignment, and filtering. Lipids were identified by matching precursor ion m/z values and product ion patterns against the LipidSearch database. Data were normalized to total peak area, log-transformed, and Pareto-scaled prior to statistical analysis. The Benjamini–Hochberg procedure was applied for *t*-tests, and paired *t*-tests were used for between-group comparisons. PLS-DA was selected for its suitability in supervised multivariate analysis of high-dimensional lipidomics data, as it maximizes group separation while identifying the most discriminating lipid species. Analysis was conducted using the mixOmics package in R and visualized with ggplot2. Key lipids distinguishing the groups were defined by *p* < 0.05 and variable importance in projection (VIP) > 1. A clustering heatmap of differential lipids was generated with the pheatmap package in R.

### 2.7 Statistical analysis

Statistical significance was assessed by independent samples *t*-test for comparisons between two groups (CM1 and NM) and one-way ANOVA for comparisons among multiple-group (CM2, FNKM1 and FNKM2). Differences were considered significant at *p* < 0.05, highly significant at *p* < 0.01, and extremely significant at *p* < 0.001. For one-way ANOVA, Duncan’s test and Dunnett’s T3 test were used for *post hoc* comparisons. Data are expressed as mean ± standard deviation (mean ± SD). Bar graphs were created with GraphPad Prism 8.

## 3 Results

### 3.1 Effects of 3% *N. haitanensis* and composite seaweed feeds on growth performance in large yellow croaker

As shown in **Table 3** (mean ± SD), the overall growth performance of large yellow croakers in the 3% *N. haitanensis* powder addition group (NM group) was better than that of the control group (CM1 group). Compared with the CM1 group, the terminal weight, weight gain rate (WGR), and specific growth rate (SGR) of large yellow croakers in the NM group increased by 1.03-, 1.11-, and 1.08-fold respectively, although the differences were not significant (*p* > 0.05). The condition factor and hepatopancreas index (HSI) of the NM group were significantly lower than those of the CM1 (*p* < 0.05) at 88.07% and 88.53%, respectively. Moreover, the caudal peduncle length and widths of the NM group were significantly higher than those of the CM1 group (*p* < 0.05). During the experimental period, the mortality rate (not shown) of large yellow croakers was extremely low due to the low water temperature.

**Table 3:** Effects of 3% *Neoporphyra haitanensis* powder and composite seaweed feed on the growth performance of large yellow croaker (*Larimichthys crocea*).

Group	CM1	NM	CM2	FNKM1	FNKM2
Initial weight (g)	200.00 ± 16.8^A^	200.00 ± 16.8^A^	213.25 ± 14.77^a^	213.25 ± 14.77^a^	213.25 ± 14.77^a^
Terminal weight (g)	284.66 ± 7.86^A^	293.75 ± 15.13^A^	335.65 ± 25.21^b^	341.03 ± 17.29^b^	415.92 ± 49.34^a^
Body length (cm)	25.85 ± 1.22^A^	27.11 ± 0.99^A^	27.61 ± 0.87^b^	28.20 ± 0.83^b^	30.34 ± 1.49^a^
Body height (cm)	7.63 ± 0.22^B^	7.95 ± 0.29^A^	8.24 ± 0.16^a^	8.23 ± 0.27^a^	8.52 ± 0.41^a^
Caudal peduncle length (cm)	6.00 ± 0.59^B^	6.73 ± 0.38^A^	6.37 ± 0.18^b^	6.76 ± 0.30^b^	7.28 ± 0.55^a^
Caudal peduncle height (cm)	1.88 ± 0.11^B^	2.03 ± 0.04^A^	2.02 ± 0.07^b^	2.05 ± 0.13^ab^	2.17 ± 0.09^a^
Body length/height	3.39 ± 0.13^A^	3.41 ± 0.10^A^	3.35 ± 0.07^b^	3.43 ± 0.10^b^	3.56 ± 0.11^a^
Caudal peduncle length/height	3.18 ± 0.27^A^	3.31 ± 0.23^A^	3.16 ± 0.14^a^	3.30 ± 0.17^a^	3.35 ± 0.19^a^
Weight gain rate (WGR, %)	42.33 ± 3.93^A^	46.88 ± 7.56^A^	57.40 ± 11.82^b^	59.92 ± 8.11^b^	95.04 ± 23.14^a^
Specific growth rate (SGR, %/day)	0.63 ± 0.05^A^	0.68 ± 0.09^A^	0.75 ± 0.12^b^	0.78 ± 0.09^b^	1.10 ± 0.20^a^
Condition factor (g/cm^3^)	1.54 ± 0.19^A^	1.36 ± 0.14^B^	1.49 ± 0.04^a^	1.40 ± 0.16^a^	1.37 ± 0.10^a^
Viscerosomatic index (VSI, %)	7.21 ± 1.42^A^	8.34 ± 2.69^A^	6.47 ± 0.64^a^	6.19 ± 0.57^a^	6.94 ± 0.60^a^
Hepatopancreas index (HSI, %)	1.61 ± 0.36^A^	1.42 ± 0.37^B^	1.21 ± 0.21^a^	1.06 ± 0.28^a^	0.95 ± 0.15^a^
Mortality rate (%)	0	0	13.5	11.5	9.5

Weight gain rate (WGR, %) = (W_t_ − W_0_)/W_0_ × 100%; Specific growth rate (SGR, % per day) = (lnW_t_ − lnW_0_)/t × 100%, where W_0_, W_t,_ and t represent initial weight, terminal weight, and feeding days, respectively; Condition factor (CF, g/cm^3^) = (W_t_/body length³) × 100%. Data are presented as mean ± SD with 30 replicates (n = 30). Viscerosomatic index (VSI, %) = viscera weight/body weight × 100%; Hepatopancreas index (HSI, %) = liver weight/body weight × 100%. Data are presented as mean ± SD with nine replicates (n = 9). CM1, CM2 groups: control group fed with the commercial feed; NM group: single seaweed group fed with feed supplemented with 3% *Neoporphyra haitanensis* powder; FNKM1 group: composite seaweed group fed with feed supplemented with 1% kelp, 1% *N. haitanensis*, and 0.1% fucoidan; FNKM2 group: composite seaweed group fed with feed supplemented with 2% kelp, 2% *N. haitanensis*, and 0.2% fucoidan. An independent samples *t*-test was used to compare the CM1 and NM groups, while one-way ANOVA was applied to compare the CM2, FNKM1 and FNKM2 groups. Uppercase letters indicate significant differences between CM1 and NM groups, while lowercase letters indicate significant differences among CM2, FNKM1 and FNKM2 groups.

In comparison, the high-concentration composite seaweed feed (FNKM2) had more significant growth performance-promoting effects in large yellow croakers. The terminal weight, WGR, and SGR of the FNKM2 group rose by 1.24-, 1.66-, and 1.47-fold, respectively, compared with the control group (CM2 group) (*p* < 0.05), with significant increases also observed in body length, body length/height, caudal peduncle length and caudal peduncle width. This revealed improvements in morphology. The terminal weight, WGR, and SGR of large yellow croakers in the low-concentration composite seaweed feed (FNKM1) group increased by 1.02-, 1.04-, and 1.04-fold, respectively, compared with the CM2 group, but the differences were not significant (*p* > 0.05). In addition, the two types of composite seaweed feed effectively decreased the large yellow croaker mortality rate under high temperature conditions during the summer, with the mortality rate in the FNKM2 group declining to 9.5% compared to 13.5% in the CM2 group.

### 3.2 Effects of different feeds on lipid content and lipid metabolism-related gene expression in large yellow croaker muscle

As shown in **Figure 1A**, the feed supplemented with 3% *N. haitanensis* powder did not significantly affect crude lipid, T-CHO, or HDL-C levels (*p* > 0.05) in large yellow croaker muscles but decreased the LDL-C level by 26.29% (*p* < 0.001). Regarding lipid metabolism-related genes, no significant differences were detected in the expression levels of *aco*, *cpt1*, or *pparα* compared with the CM1 group (*p* > 0.05), whereas *pgc-1α* expression was significantly upregulated by 3.08-fold (*p* < 0.001). In contrast, the expressions of *lpl*, *fas* and *srebp1* were significantly downregulated, by approximately 39.24%–57.60% compared with those in the CM1 group (*p* < 0.001; **Figure 1B**). Compared with single seaweed feed, the composite seaweed feeds induced more pronounced changes in the lipid composition of large yellow croaker muscle (**Figure 2A**). Both composite seaweed feeds significantly decreased the crude lipid and LDL-C levels in large yellow croaker muscles, which declined by 27.87% and 17.88% in the FNKM1 group, respectively, and by 24.20% and 27.57% in the FNKM2 group, respectively (*p* < 0.05). Moreover, compared with the CM2 group, the two composite seaweed feed groups significantly increased HDL-C levels by 1.46- and 1.33-fold, respectively (*p* < 0.05), but had no significant effects on T-CHO (*p* > 0.05). With regard to lipid metabolism-related genes, composite seaweed feeds significantly enhanced the expressions of *cpt1*, *pparα*, *pgc-1α* and *lpl* (**Figure 2B**). In particular, *pgc-1α*, expression increased by 4.94- and 9.87-fold in the FNKM1 and FNKM2 groups, respectively. No significant differences were observed in the expression levels of *fas* and *srebp1* (*p* > 0.05).

**Figure 1:**
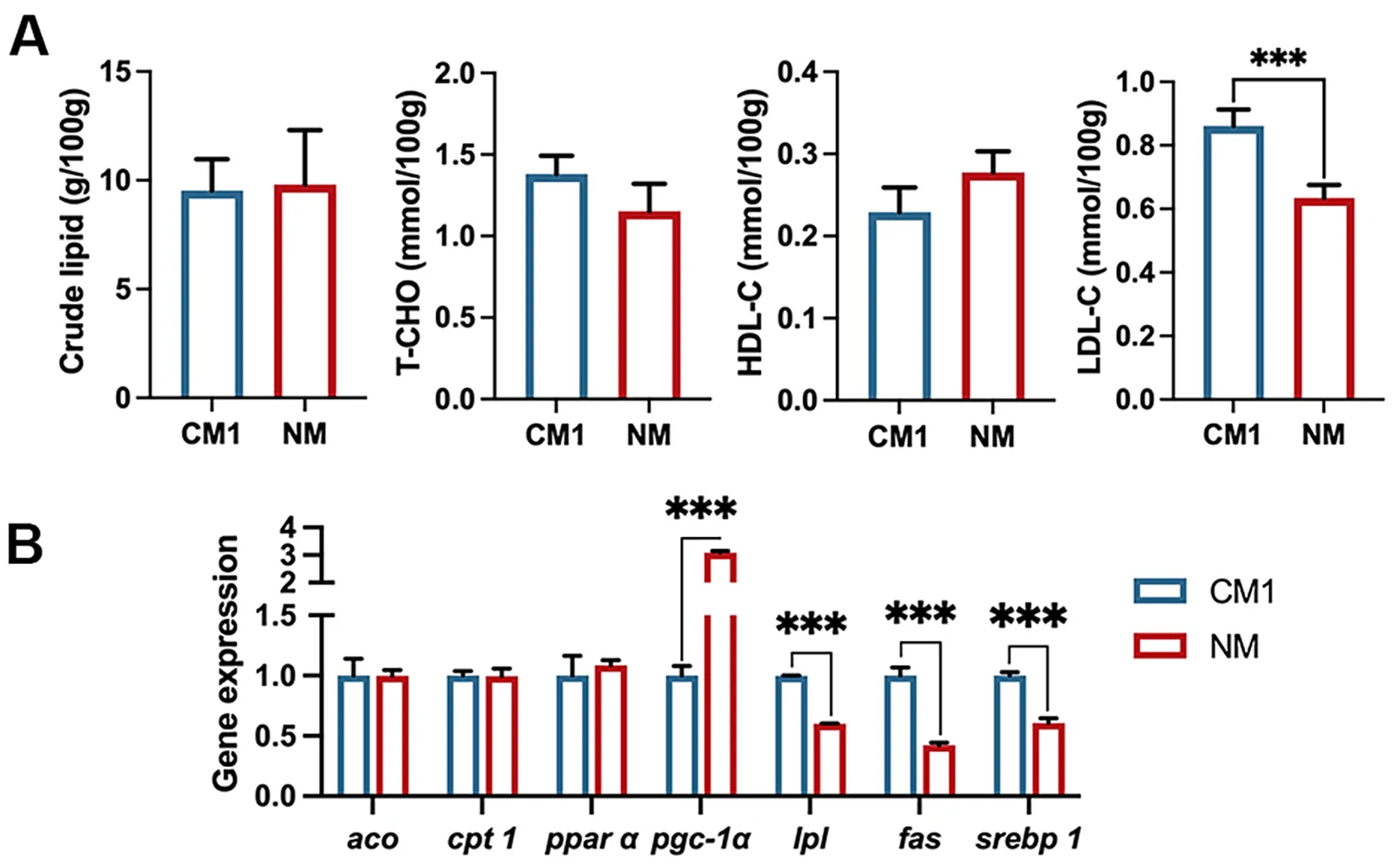
Effects of 3% *Neoporphyra haitanensis* powder on large yellow croaker (*Larimichthys crocea*) muscle crude fat, cholesterol, and lipid metabolism gene expression (n = 3). **(A)** Effects of 3% *N. haitanensis* powder on large yellow croaker muscle crude fat, total cholesterol (T-CHO), high-density lipoprotein cholesterol (HDL-C), and low-density lipoprotein cholesterol (LDL-C) levels. **(B)** Effects of 3% *N. haitanensis* powder on large yellow croaker lipid metabolism gene expression. *aco*: acyl-CoA oxidase*; cpt1*: carnitine palmitoyltransferase 1; *pparα*: peroxisome proliferator-activated receptor *α; pgc-1α:* peroxisome proliferator-activated receptor γ coactivator 1α*; lpl*: lipoprotein lipase; *fas:* fatty acid synthase; *srebp1*: sterol regulatory element-binding protein 1. “***” indicates an extremely significant difference between two groups (*p* < 0.001). CM1 group, control group fed with the commercial feed; NM group, single seaweed group fed with feed supplemented with 3% *N. haitanensis* powder.

**Figure 2:**
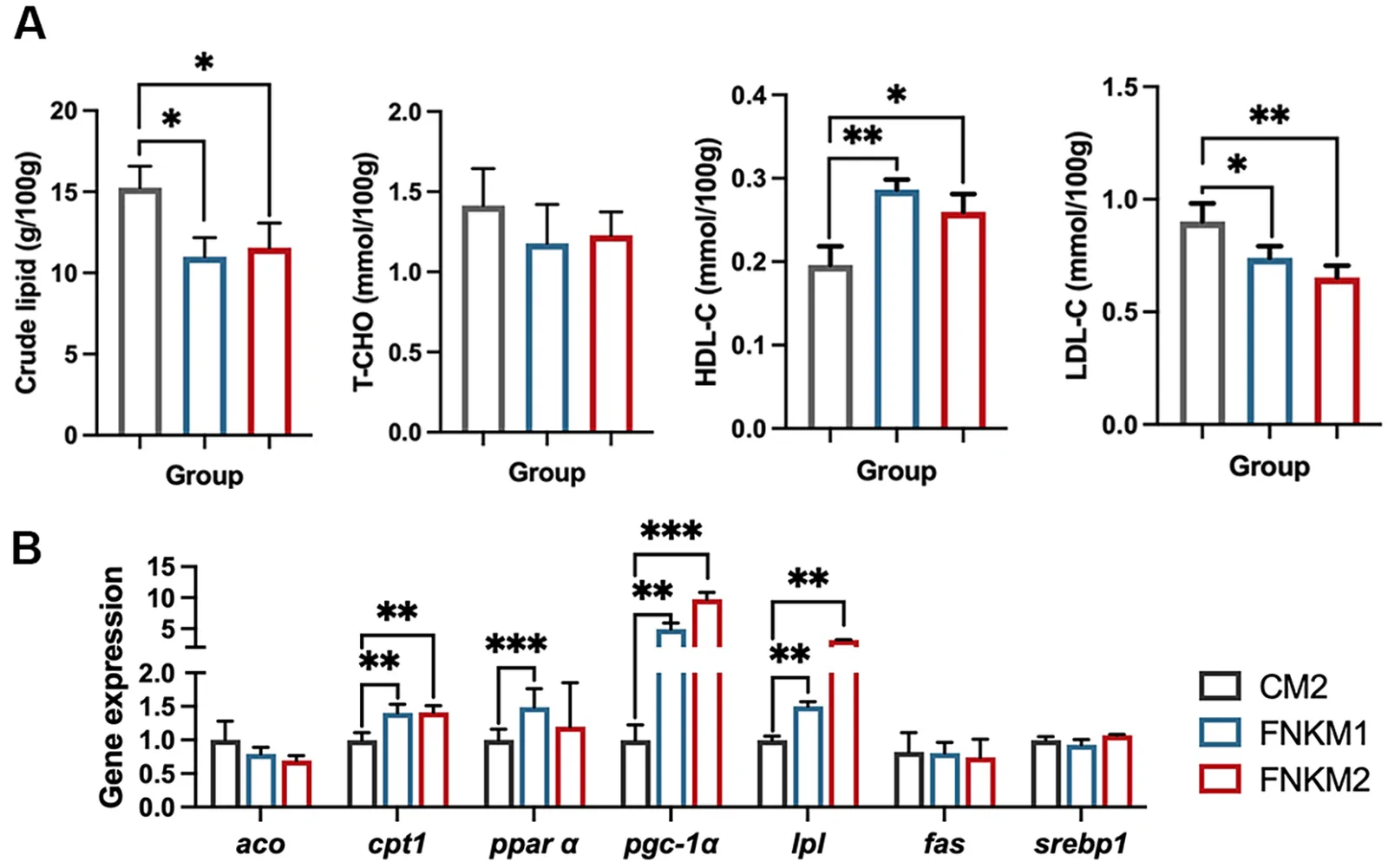
Effects of composite seaweed feed on large yellow croaker (*Larimichthys crocea*) muscle crude fat, cholesterol, and lipid metabolism gene expression (n = 3). **(A)** Effects of composite seaweed feed on large yellow croaker muscle crude fat, total cholesterol (T-CHO), high-density lipoprotein cholesterol (HDL-C), and low-density lipoprotein cholesterol (LDL-C) levels. **(B)** Effects of composite seaweed feed on large yellow croaker lipid metabolism gene expression. *aco*: acyl-CoA oxidase*; cpt1*: carnitine palmitoyltransferase 1; *pparα*: peroxisome proliferator-activated receptor α; *pgc-1α*: peroxisome proliferator-activated receptor γ coactivator 1α; *lpl*: lipoprotein lipase; *fas*: fatty acid synthase; *srebp1*: sterol regulatory element-binding protein 1. “*”, “**”, and “***” represent significant differences at *p* < 0.05, *p* < 0.01, and *p* < 0.001, respectively. CM2 group, control group fed with the commercial feed; FNKM1 group, composite seaweed group fed with feed supplemented with 1% kelp, 1% *N. haitanensis*, and 0.1% fucoidan; FNKM2 group, composite seaweed group fed with feed supplemented with 2% kelp, 2% *N. haitanensis*, and 0.2% fucoidan.

### 3.3 Lipidomic analysis of large yellow croaker muscle fed with different seaweed feeds

To investigate the effects of different seaweed feeds on the lipid composition in large yellow croaker muscle, UPLC-MS analysis was performed. The results showed that the feed supplemented with 3% *N. haitanensis* powder significantly altered the lipid composition of large yellow croaker muscles (**Figure 3A**). The detailed information of lipids was listed in **Supplementary Table 1**. In total, 154 differentially abundant lipids (VIP > 1.0 and *p* < 0.05) were identified in the NM group, of which 133 were significantly upregulated and 21 were significantly downregulated. As shown in **Figure 3B**, the top 15 differentially abundant lipids were phospholipids, and their abundance in the NM group was significantly increased. These phospholipids included nine lysophosphatidylcholines (LPCs) and three lysophosphatidylethanolamines (LPEs), with their abundances increasing by approximately 3.11–27.03-fold. These lipids mainly contained unsaturated fatty acid chains, among which LPC 22:6 and LPE 20:3 displayed the highest relative abundances, increasing by 13.33- and 6.94-fold, respectively, compared with the CM1 group. The clustered heat map (**Figure 3C**) revealed that 44 of the top 50 differentially abundant lipids were significantly upregulated in the NM group, whereas six were downregulated. The downregulated lipids included five triglyceride (TG) species, such as TG (16:0_18:2_21:0), TG (16:1_10:0_16:1), and TG (16:1_12:0_14:0), whose abundances decreased by 34.64%–61.08% compared with the CM1 group. These TGs mainly contained C16 fatty acid chains, with a predominance of saturated fatty acids. In contrast, the lipids with increased abundances were mainly LPCs, along with phosphatidylcholine (PC), LPE, and diglyceride (DG). Overall, *N. haitanensis* supplementation was associated with decreased abundances of TGs rich in saturated fatty acid chains and increased abundances of phospholipids containing unsaturated fatty acids.

**Figure 3:**
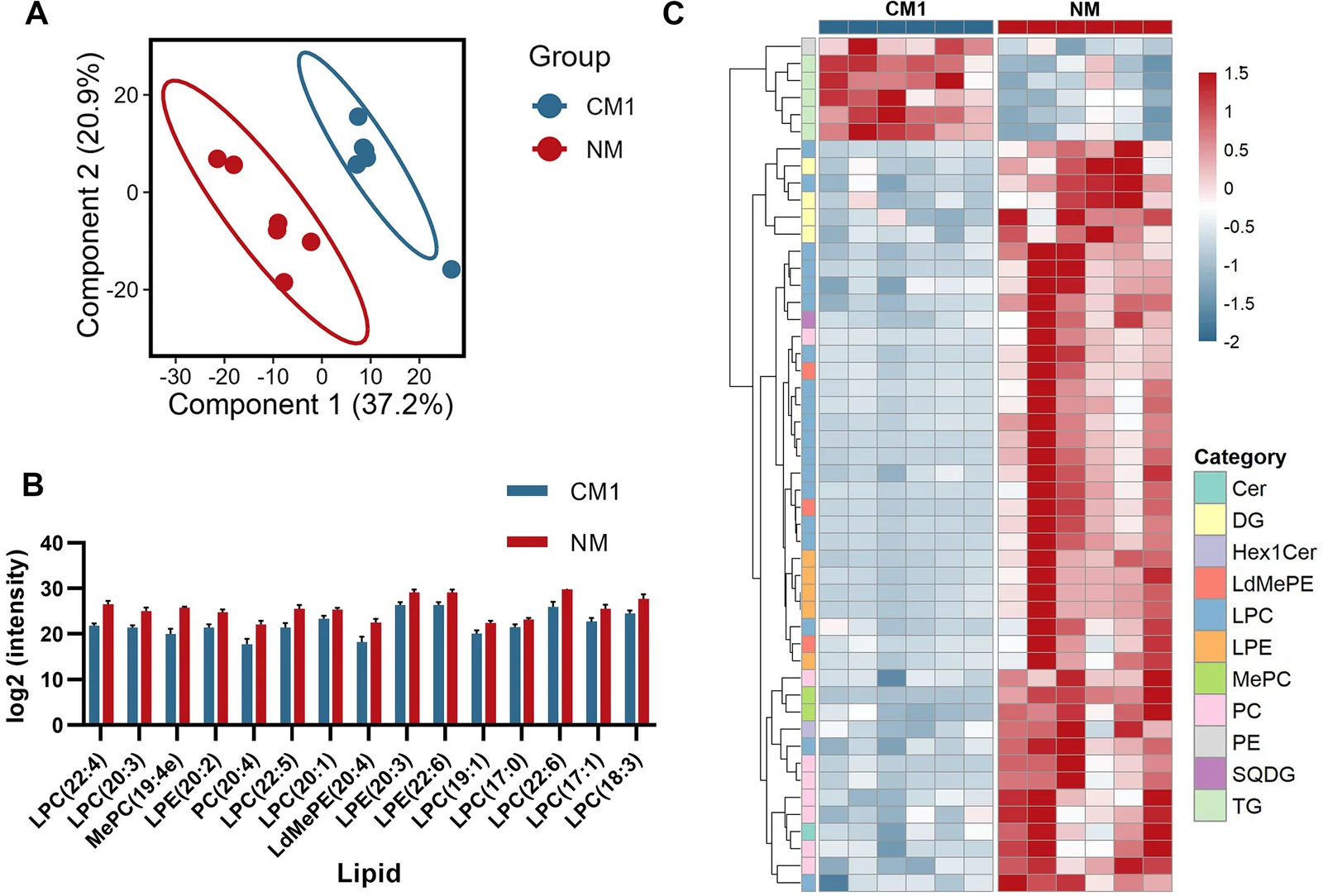
Effects of 3% *Neoporphyra haitanensis* powder on large yellow croaker (*Larimichthys crocea*) muscle lipid metabolome (n = 6). **(A)** Partial least squares discriminant analysis (PLS-DA) score map of large yellow croaker lipid metabolome. **(B, C)**. **(B)** Histogram of top 15 differentially abundant lipids in large yellow croaker muscles and **(C)** clustered heat map of top 50 differentially abundant lipids. Cer, ceramide; DG, diglyceride; Hex1Cer, hexosylceramide; dMePEs, methylphosphatidylethanolamine; LPC, lysophosphatidylcholine; LPE, lysophosphatidylethanolamine; MePC, methylphosphatidylcholine; PC, phosphatidylcholine; PE, phosphatidylethanolamine; SQDG, sulfoquinovosyl diacylglycerol; TG, triglyceride; LdMePE, Lyso-dimethylphosphatidylethanolamine. CM1 group: control group fed with the commercial feed; NM group: single seaweed group fed with feed supplemented with 3% *N. haitanensis* powder.

The effects of composite seaweed feed on the lipid composition of large yellow croaker muscle were further evaluated (see **Supplementary Table 2** for lipid identification). The PLS-DA result showed clear separation between the FNKM1 and CM2 group, whereas partial overlap was observed between the FNKM2 and CM2 group (**Figure 4A**). Based on the identification criteria (VIP > 1.0 and *p* < 0.05), 156 and 98 differentially abundant lipids were identified in the FNKM1 and FNKM2 groups, respectively, compared with the CM2 group. Among them, 55 were upregulated and 101 were downregulated in the FNKM1 group, whereas 23 were upregulated and 75 were downregulated in the FNKM2 group. As shown in **Figure 4B**, most of the top 10 differentially abundant lipids in the FNKM1 group were phospholipids, including four phosphatidylserines (PSs), three methylphosphatidylcholines (MePCs), two phosphatidylglycerols (PGs), and one phosphatidylethanolamine (PE). These lipids showed decreased abundances, ranging from approximately 30.22% to 88.15%. In the FNKM2 group, nine of the top 10 differentially abundant lipids showed a downregulation trend, including PS (18:0_20:5), PC (22:4_18:2), SM (t40:0), and two TGs (TG 16:0_9:0_9:0 and TG 20:3e_18:1_18:2) (**Figure 4C**). Only one TG (TG 18:4_16:0_16:0) showed an upregulation trend. Further analysis of the top 50 differentially abundant lipids (**Figure 4D**) identified a total of 29 downregulated lipids in the FNKM1 group, including six PSs, five PEs, four TGs, and four MepCs; while 21 lipids were upregulated, including seven PSs and five TGs. In the FNKM2 group, 44 of the top 50 differentially abundant lipids were downregulated (**Figure 4E**), including nine TGs, six PCs, six PSs, and six ceramides (Cer), whereas only six were upregulated, including three TGs, two PCs, and one wax esters (WE). Overall, in contrast to the NM group, in which most differentially abundant lipids exhibited increased abundance, most differentially abundant lipids in the muscle of large yellow croakers fed the composite seaweed diet exhibited decreased abundance. This pattern was more pronounced in the FNKM2 group, which had a higher proportion of downregulated lipids.

**Figure 4:**
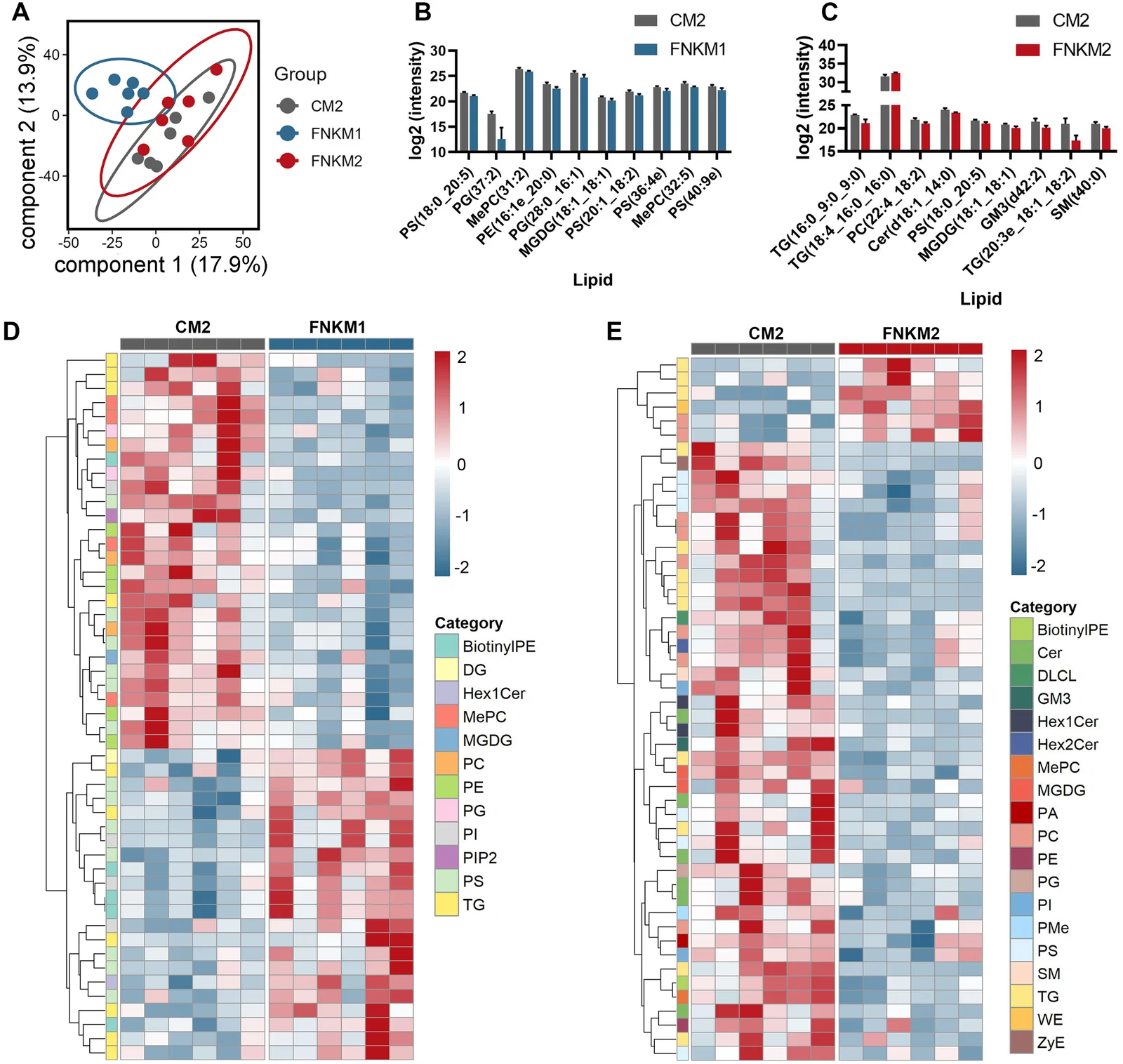
Effects of composite seaweed feed on large yellow croaker (*Larimichthys crocea*) muscle lipid composition and lipid metabolism-related gene expression (n = 6). **(A)** Partial least squares discriminant analysis (PLS-DA) score map of large yellow croaker lipid metabolome. **(B, C)**. Histogram of top 10 differentially abundant lipids in large yellow croaker muscles in **(B)** FNKM1 and **(C)** FNKM2 group. **(D, E)**. Clustered heat map of top 50 differentially abundant lipids in large yellow croaker muscles in **(D)** FNKM1 and **(E)** FNKM2 group. PS, phosphatidylserine; PG, phosphatidylglycerol; MEPC, methylphosphatidylcholine; PE, phosphatidylethanolamine; MGDG, monogalactosyl diglyceride; TG, triglyceride; Cer, ceramide, GM3, monosialodihexosylganglioside; SM, sphingomyelin; Biotinyl PE, biotinlyated phosphatidylethanolamine; DG, diglyceride; Hex1Cer, hexosylceramide; PC, phosphatidylcholine; PI, phosphatidylinositol; PIPa, phosphatidylinositol-4, 5-bisphosphate; DLCL, cardiolipin antibody; Hex2Cer, dihexosylceramide; PA, phosphatidic acid; PME, phosphatidylmethanol; WE, wax esters; ZyE, zymosterol. CM2 group: control group fed with the commercial feed; FNKM1 group: composite seaweed group fed with feed supplemented with 1% kelp, 1% *N. haitanensis*, and 0.1% fucoidan; FNKM2 group: composite seaweed group fed with feed supplemented with 2% kelp, 2% *N. haitanensis*, and 0.2% fucoidan.

### 3.4 Effects of composite seaweed feed on immune function in large yellow croaker

Compared with the CM2 group, the composite seaweed diet groups showed significant differences in the serum activities of immune- and antioxidant-related enzymes, as well as in the hepatic expression of inflammatory cytokines (**Figure 5**). In the FNKM1 group, ACP, ALP, and LYS activities elevated by 1.35-, 1.44-, and 1.39-fold, respectively, compared with the CM2 group, while GSH-Px and CAT activities increased by 1.56- and 2.08-fold, respectively (**Figure 5A**; p < 0.05). In the FNKM2 group, ACP and ALP activities increased by 1.31- and 1.62-fold, respectively, and CAT activity was 1.93-fold that of the CM2 group, whereas LYS activity declined by 41.72% (p < 0.05). No significant differences were observed in GSH-Px or SOD activity relative to the CM2 group (p > 0.05). In the FNKM1 group, the expressions of anti-inflammatory factors-related genes were generally comparable to those in the CM2 group, with only il-4 showing a significant decrease of 31.25% (**Figure 5B**; p < 0.05). In contrast, the FNKM2 group showed significantly higher expression of il-4 and tgf-β, reaching 1.29- and 2.26-fold those of the CM2 group, respectively, while il-10 expression was decreased by 78.38% (p < 0.05). No significant differences in the expression of arg1 among the groups. Besides, the expressions of pro-inflammatory factor-related genes also differed among the composite seaweed feed groups. In the FNKM1 group, the expressions of il-1β and tnf-α elevated by 1.48- and 1.66-fold, respectively, compared to the CM2 group. In the FNKM2 group, these genes expressions increased by 1.16- and 2.51-fold, respectively, compared with the CM2 group (p < 0.05).

**Figure 5:**
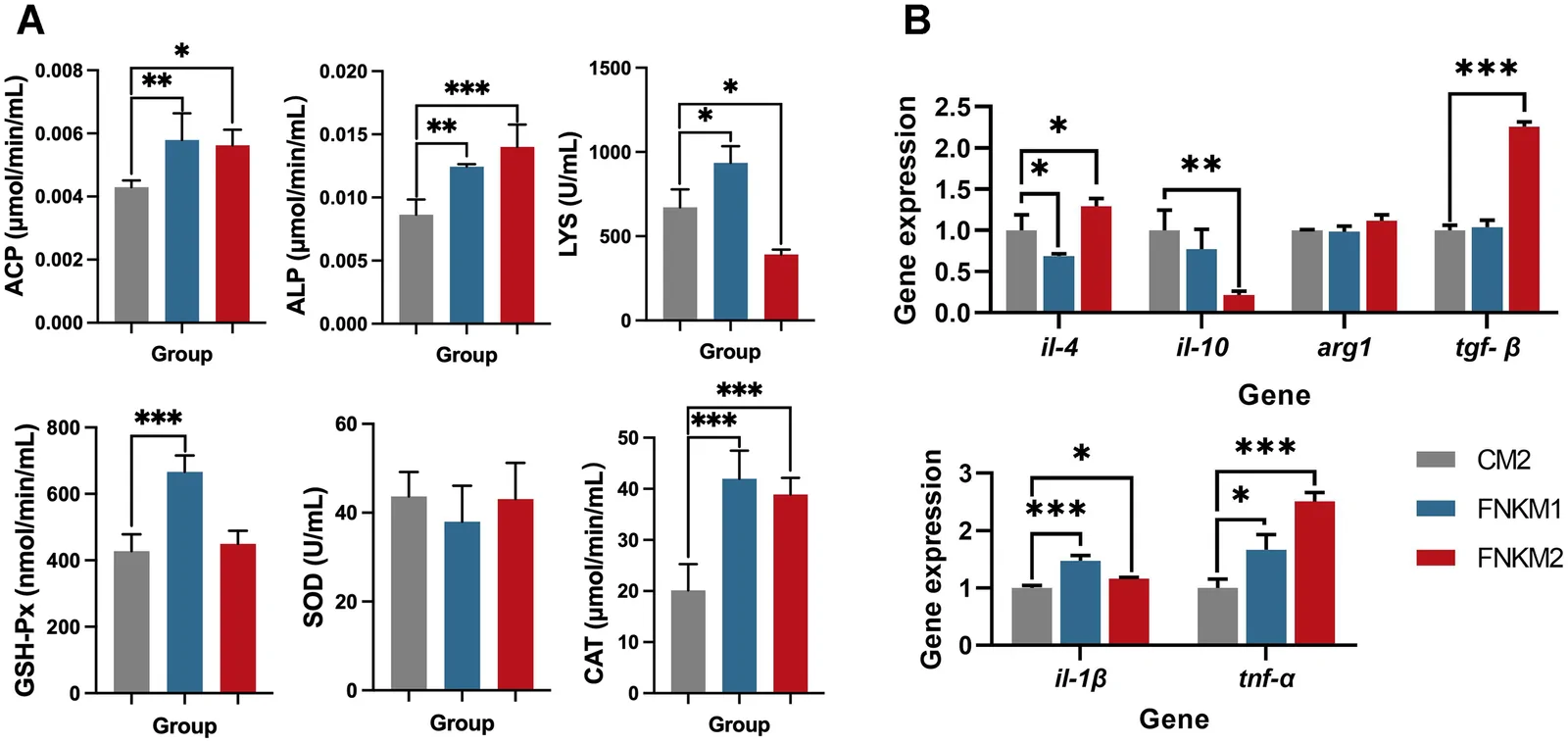
Effects of composite seaweed feed on large yellow croaker (*Larimichthys crocea*) immune performance (n = 3). **(A)** Effects of composite seaweed feed on large yellow croaker immune and antioxidant enzyme activities. ACP, acid phosphatase; ALP, alkaline phosphatase; LYS, lysozyme; GSH-Px, glutathione peroxidase; SOD, superoxide dismutase; CAT, catalase. **(B)** Effects of composite seaweed feed on large yellow croaker inflammatory cytokine gene expression levels. *il-4*: interleukin 4; *il-10*: interleukin-10; *arg1*: arginase-1; *tgf-β*: transforming growth factor β; *il-1β*: interleukin-1β; *tnf-α*: tumor necrosis factor α. “*”, “**”, and “***” represent significant differences at *p* < 0.05, *p* < 0.01, and *p* < 0.001, respectively. CM2 group: control group fed with the commercial feed; FNKM1 group: composite seaweed group fed with feed supplemented with 1% kelp, 1% *N. haitanensis*, and 0.1% fucoidan; FNKM2 group: composite seaweed group fed with feed supplemented with 2% kelp, 2% *N. haitanensis*, and 0.2% fucoidan.

### 3.5 Integrated analysis of growth performance, lipid metabolism, and immune responses to single and composite seaweed feeds

Combining the data from our previous study (Zhu et al., 2025) on 0.2% fucoidan (FM) and 3% kelp powder (KM) with the data for the NM, FNKM1, and FNKM2 groups in the present study, the effects of single and composite seaweed feeds were examined by comparing the fold changes of each seaweed feed relative to its corresponding control group. For growth performance, the WGR of large yellow croaker was significantly higher only in the FNKM2 group (*p* < 0.05, **Figure 6A**). For lipid-related parameters, both FNKM1 and FNKM2 significantly decreased the crude lipid content in the muscle and increased HDL-C level. LDL-C levels were reduced in all feed groups, with the largest declines observed in the KM group. And the greatest reduction in T-CHO was observed in the FM group. With regard to lipid metabolism-related gene expression, the composite seaweed feeds showed greater increases associated with fatty acid oxidation-related genes. Moreover, the high-concentration composite seaweed feed group (FNKM2) generally showed stronger effects than the low-concentration group (FNKM1). In contrast, lipid synthesis-related genes exhibited a downregulation trend in the single seaweed feed groups, with greater changes than those observed in the composite seaweed feed groups. An integrated analysis of significantly changed TGs, phospholipids and EPA/DHA-containing phospholipids in the muscle lipidome also revealed that several TGs displayed increased abundances in the FM group, whereas some TGs decreased in the KM and NM groups. The reduction in TG was more pronounced in the FNKM1 group than in the single seaweed feed groups, whereas the FNKM2 group showed a negative effect, with increased abundances observed for some TGs. In addition, only the NM group showed significant increases in phospholipids and EPA/DHA-containing phospholipids, while these lipids generally exhibited decreasing trends in the KM, FNKM1, and FNKM2 groups, with only a slight increase in EPA/DHA-containing phospholipids observed in the FNKM2 group.

**Figure 6:**
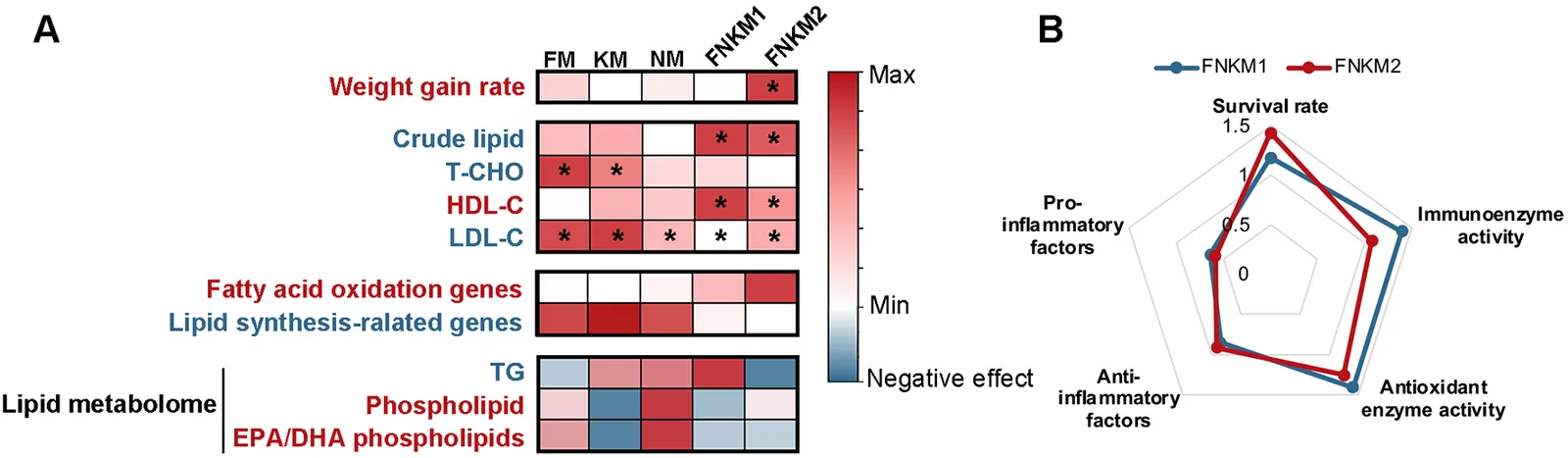
**(A)**. Heat map of effects of single- and composite seaweed feeds on large yellow croaker growth and muscle lipid composition. Color intensity represents the degree of improvement, where a darker red indicates positive effects, while blue represents negative effects. Left indicator text color: red indicates positive indicators, while blue represents negative indicators. For each treatment group, the relative change was calculated relative to its corresponding control group, and the values of each indicator were normalized accordingly. The positive indicator normalization formula was (*x* − *x*_min_)/(*x*_max_ − *x*_min_) × 100, while the negative indicator normalization formula was (*x*_max_ − *x*)/(*x*_max_ − *x*_min_) × 100. In the heat map, “*” indicates a significant difference compared with the control group (*p* < 0.05). The relative rate of change for lipid metabolism genes and metabolome indicators was calculated by summing the individual differences (significance not shown). **(B)** Radar chart of the effects of different concentrations of composite seaweed feed on large yellow croaker survival rate, serum biochemistry and liver immunity. Improvement index (IoI) = mean of treatment group/mean of control group (positive indicators), IoI = mean of control group/mean of treatment group (negative indicators); the integrated improvement index (∑IoI) is the geometric mean of the various IoIs, where a higher value indicates better overall improvement results. FM group; single seaweed group fed with feed supplemented with 0.1% fucoidan; KM group: single seaweed group fed with feed supplemented with 3% kelp powder; NM group: single seaweed group fed with feed supplemented with 3% *N. haitanensis* powder; FNKM1 group: composite seaweed group fed with feed supplemented with 1% kelp, 1% *N. haitanensis*, and 0.1% fucoidan; FNKM2 group: composite seaweed group fed with feed supplemented with 2% kelp, 2% *N. haitanensis*, and 0.2% fucoidan. FM and KM data are obtained from Zhu et al., 2025.

To characterize the responses to the two composite seaweed feeds concentrations, the improvement index (IoI) was calculated for individual survival, immune- and antioxidant-related enzyme activities, and inflammatory cytokines-related gene expressions, and the corresponding integrated improvement index (∑IoI) was presented as a radar chat (**Figure 6B**). The FNKM2 group showed a higher survival-related ∑IoIs with the value of 1.42. For immune and antioxidant-related enzyme activities, the ∑IoI values of the FNKM1 group were both greater than 1, whereas the ∑IoI values of inflammatory cytokines were similar between FNKM1 and FNKM2.

## 4 Discussion

Seaweed based feed supplementation have attracted increasing attention in aquaculture because of its potential to improve growth performance and modulate metabolic and immune responses in fish. Different seaweed species contain diverse nutrients and bioactive compounds, and their combined use may provide complementary nutritional and functional characteristics that promote fish growth and development, enhance lipid metabolism regulation, and improve health status (García-Márquez et al., 2023; Sattanathan et al., 2023; Seyedalhosseini et al., 2023; Zhang et al., 2025). In the present study, the responses of large yellow croaker to single and composite seaweed feeds were evaluated on growth performance, lipid metabolism, and immune responses. Because the two feeding trials were conducted independently during different experimental periods, direct statistical comparisons between the two experiments were not performed. Instead, fold changes relative to the corresponding control group were used for descriptive comparison of the responses to different seaweed feeds across the two independent experiments.

### 4.1 Composite seaweed feed promotes the growth of large yellow croaker

Seaweeds are rich in high-quality proteins, polyunsaturated fatty acids, polysaccharides, and a balanced amino acid profile that can effectively support protein synthesis in fish, thereby promoting growth (Harnedy and FitzGerald, 2011). Previous studies have shown that combinations of different algal ingredients can affect fish growth and health status (García-Márquez et al., 2023; Seyedalhosseini et al., 2023). For example, in red seabream (*P. major*), supplementation with a composite algal powder containing 20% *Nannochloropsis* and 5% *Schizochytrium* or further supplementation with 20% *Chlorella*, increased growth rate, whereas supplementation with a single alga did not produce significant effect (Seong et al., 2021). In the present study, our findings align with these observations. Supplementation with 3% *N. haitanensis* powder alone, as well as 3% kelp powder or 0.2% fucoidan feed (Zhu et al., 2025), did not significantly promote growth in large yellow croaker. In contrast, the composite seaweed feed consisting of 2% *N. haitanensis* powder, 2% kelp powder, and 0.2% fucoidan (FNKM2) significantly enhanced growth, with a 1.66-fold increase in WGR compared with the control group. Thus, the higher growth performance observed in the FNKM2 group indicated that the specific combination and concentration of seaweed ingredients used in FNKM2 were associated with greater improved growth performance under the experimental conditions of the present study. This synergy likely arises from complementary nutrient profiles among algal species (Ferreira et al., 2021). *N. haitanensis* is rich in protein (>35% dry weight) and contains abundant EPA, which may provide structural and energetic support for growth (Liang et al., 2022). Kelp contains bioactive compounds, including fucoidan, alginate, mannitol, and polyphenols, while fucoidan has been reported to influence digestion, intestinal health, immunity responses, and metabolic regulation in aquaculture species (Cui et al., 2019; Mahgoub et al., 2020; Salehpour et al., 2021). The combination of these seaweed ingredients may therefore provide a broader range of nutrients and bioactive compounds than a single seaweed ingredient. Furthermore, a concentration-dependent response was also observed within the composite-seaweed feeding trail. The low-concentration composite seaweed feed (FNKM1) showed limited growth performance, suggesting that a certain threshold dose is required to maximize the beneficial effects of seaweed additives. A similar concentration-dependent response was reported in *Labeo rohita* fed a composite seaweed feed containing *Chlorella vulgaris*, *Euglena viridis*, and *Spirulina platensis*, in which significant growth promotion was observed at a total amount of seaweed powder concentration of 0.2%, whereas no significant effect was observed at 0.1% (Sattanathan et al., 2023). Therefore, the present findings suggested that a specific supplementation level of the composite seaweed feed, represented by FNKM2, may be beneficial for the growth of large yellow croaker.

### 4.2 Single and composite seaweed feeds alter lipid metabolism in large yellow croaker

Lipid metabolism in fish involves coordinated processes of lipid synthesis, hydrolysis, transport, oxidation, and storage. In muscle, these processes are reflected at the biochemical level by changes in crude lipid and cholesterol-related parameters and at the lipidomic level by changes in neutral lipids, particularly TGs, and polar lipids such as phospholipids. In this present study, both FNKM1 and FNKM2 significantly decreased muscle crude lipid content and LDL-C levels while increasing HDL-C levels relative to the corresponding CM2 control group, whereas single seaweed feeds showed no significant changes in crude lipids compared with the corresponding control group (Zhu et al., 2025). The results suggested that the composite seaweed feeds effectively regulated lipid metabolism in large yellow croaker, reducing excessive lipid deposition in muscle. The effects may be related to the different nutritional and bioactive properties of kelp, *N. haitanensis*, and fucoidan. Studies have shown that kelp and its bioactive compound fucoidan may downregulate SREBP-2/HMGCR to inhibit cholesterol synthesis, while concurrently upregulating LDL receptors or CYP7A1 to enhance cholesterol excretion (Park et al., 2016; Yokota et al., 2016; Zhong, 2016). Polysaccharides from *N. haitanensis* have been shown to reduce T-CHO and LDL-C levels in mice (Chen, 2021). These findings indicated that composite seaweed feeds systematically modulate lipid metabolic networks in animals through multi-component synergistic effects, achieving integrated regulation of a systemic lipid-lowering benefits.

Muscle lipids in fish consist of neutral lipids, particular TGs, which represent an important form of energy storage, and polar lipids (mainly phospholipids) which are major structural component of cellular membranes (Frøyland et al., 1998). In our previous study, kelp powder broadly downregulated lipids content, particularly TGs and phospholipids containing unsaturated fatty acyl chains, whereas fucoidan had relatively limited effects (Zhu et al., 2025). However, reduced phospholipid levels may compromise membrane integrity, adversely affecting fish quality, taste, and nutritional value (Li et al., 2022; Morita and Ikeda, 2022). In contrast, *N. haitanensis* powder in the present study was associated with decreased TGs levels but increased phospholipid abundance. This difference suggests that different seaweed ingredients may have distinct effects on muscle lipid remodeling. Composite seaweed feeds exhibited combined regulatory features. FNKM1 resulted in a more pronounced reduction in TG content than single seaweed feeds, but also decreased the abundance of some phospholipids. In FNKM2, most differentially abundant lipids were downregulated, whereas total TG abundance increased. These findings possibly due to compensatory TG synthesis triggered by stronger lipid-lowering effects. Similar compensatory responses have been reported in gilt-head bream (*Sparus aurata*), where 1% microalgal supplementation significantly increased muscle TG accumulation, whereas 0.5% did not (Perera et al., 2020).

TG homeostasis is maintained through coordinated regulation of lipid synthesis and degradation (Wunderling et al., 2023). LPL catalyzes TG hydrolysis to release fatty acids for energy production (Kersten, 2014), whereas SREBP-1 promotes fatty acid synthesis via *acc* and *fas* (Shimano, 2002). PGC-1α cooperates with PPARα to promote fatty acid oxidation-related gene expression, including *cpt1* (Cao et al., 2026). In single seaweed feed groups, reduced TG content was accompanied by downregulation of *lpl*, *srebp-1*, and *fas*, indicating adaptive suppression of both TG hydrolysis and fatty acid synthesis (Zhu et al., 2025). In composite seaweed feed groups, *pgc-1α* was markedly upregulated (FNKM1: 4.94-fold; FNKM2: 9.87-fold), exceeding single seaweed feed groups (Zhu et al., 2025), with similar trends for *cpt1*, *pparα* and *lpl*. These findings indicate that composite seaweed feeds, particularly FNKM1, simultaneously promoted TG hydrolysis and fatty acid oxidation while maintaining fatty acid synthesis at baseline levels, thereby achieving lipid metabolic balance and preventing excessive TG accumulation.

### 4.3 Composite seaweed feed modulates immune responses in large yellow croaker

Immune- and antioxidant-related enzyme activity data showed that composite seaweed feed significantly enhanced non-specific immune function and antioxidant defense in large yellow croakers in large yellow croakers. ACP and ALP were significantly increased in both composite seaweed feed groups, indicating activation of phagocytic immune pathways (Bune et al., 2001; Chen et al., 2025). Increased GSH-Px and CAT activities indicated enhanced antioxidant defense against oxidative damage and improved capacity to maintain cellular homeostasis (Jomova et al., 2024). These results support the potential role of seaweed supplementation as a mild immunomodulators (Mirzadeh et al., 2021). A healthy immune response requires dynamic equilibrium between pro-inflammatory and anti-inflammatory processes (Cicchese et al., 2018). In the FNKM1 group, pro-inflammatory factors (*il-1β* and *tnf-α*) were upregulated while anti-inflammatory factor (*il-4*) was downregulated, indicating that the immune system was activated and maintained in a state of vigilance (Ma et al., 2020; Sahragard et al., 2026). In the FNKM2 group, pro-inflammatory factors (*il-1β* and *tnf-α*) and certain anti-inflammatory factors (*il-4* and *tgf-β*) were simultaneously upregulated. The simultaneous upregulation of both pro- and anti-inflammatory cytokine-related genes suggests that the immunologically active components, such as seaweed polysaccharides and fucoidan, may enhance immune function by activating multiple immune pathways (Yang et al., 2014; Sattanathan et al., 2023; Sahragard et al., 2026). The elevated expression of anti-inflammatory cytokines may serve as a negative feedback mechanism that limits excessive inflammation and prevents tissue damage (Asgari et al., 2024). Given the absence of overt infection or pathological inflammation, together with the reduced mortality rates in the FNKM1 and FNKM2 groups (14.81% and 29.63% lower than that of the CM2 group, respectively), this concurrent upregulation more likely reflects immune homeostasis rather than pathological inflammation. Similar to our results, Zhao et al. (2024) also found simultaneous upregulation of pro- and anti-inflammatory cytokines in native fish exposed to phytosphingosine (PHS), a component of *Microcystis aeruginosa* exudates, and attributed this to an immunostimulatory effect with negative feedback regulation. Collectively, these results indicate that composite seaweed feeds act as immune boosters, enhancing immune function through mild immune stimulation and promoting an efficient, well-regulated immune state (Ma et al., 2020; Rajan et al., 2023).

## 5 Conclusions

This study demonstrated that single-seaweed feeds and composite seaweed feeds differentially affected the growth, lipid metabolism, and immune function of large yellow croaker. *N. haitanensis* powder effectively reduced muscle TG, T-CHO, and LDL-C while maintaining or increasing phospholipid levels, reflecting an optimized lipid profile. In contrast, composite seaweed feeds significantly decreased crude fat, TG (in the FNKM1 group), phospholipid (in the FNKM1 group), EPA/DHA-containing phospholipids, and LDL-C contents while increasing HDL-C levels, accompanied by upregulation of lipid oxidation genes (*cpt1*, *pparα*, *pgc-1α)*and the TG hydrolysis-related gene (*lpl*). The phospholipid decline in the composite feed groups may be attributable to the kelp powder component, suggesting that reducing the kelp powder proportion while increasing *N. haitanensis* powder could help maintain phospholipid balance. Furthermore, FNKM2 more effectively promoted growth performance, whereas FNKM1 exhibited stronger immunomodulatory effects, including elevated survival rate and enhanced immune and antioxidant enzyme activities. Collectively, these findings indicate that composite seaweed feeds optimize lipid metabolism and activate immune defenses through multi-component synergism, providing a scientific basis for the development of functional feeds for large yellow croaker.

## Data Availability

The original contributions presented in the study are included in the article/**Supplementary Material**. Further inquiries can be directed to the corresponding author.
